# Federalism, Science, and State Regulation of Reproduction

**DOI:** 10.1017/jme.2025.67

**Published:** 2025

**Authors:** Lindsay F. Wiley

**Affiliations:** Law, UCLA, Los Angeles, United States

**Keywords:** reproductive rights, reproductive health, reproductive justice, federalism, constitutional law, surrogacy

## Abstract

In the aftermath of the Supreme Court’s decision ending federal constitutional protection for abortion, interstate and federal-state conflicts are revealing the importance of federalism to reproductive justice. This shift has implications for health and social science research agendas because scientific evidence plays a less significant role in disputes over which government actor is empowered to regulate reproduction than it does in conflicts over reproductive rights.

Discussions about the relationship between regulation and reproduction typically focus on individual rights, but the Supreme Court’s 2022 decision eliminating constitutional protection for the right to terminate a pregnancy^1^ has triggered interstate and federal-state clashes that reveal the importance of constitutional federalism to reproductive justice.^2^ This shift has important implications for health and social science research agendas. Scientific evidence plays a much less significant role in disputes over which government actor is constitutionally empowered to regulate reproduction compared to the role scientific evidence plays in conflicts between individual rights and concerns about the negative effects of reproductive choices and technologies.

## Constitutional Federalism

It is important for health and social science researchers to understand how constitutional federalism shapes opportunities for realizing reproductive justice. In addition to setting forth individual rights and freedoms, our national constitution divides power between the federal government and the states (federalism) and among the branches of the federal government (separation of powers).^3^ Together, federalism and separation of powers impose structural constraints that determine which government actor has authority to adopt any given intervention to address a matter of public concern. The US Constitution grants limited powers to the federal government. These include the power to regulate interstate commerce and powers to tax and spend in ways that indirectly regulate individuals and businesses. The federal government also has power to enforce federal protections for individual rights, including against the states. Unlike the federal government, state governments have general power to regulate to protect the health, safety, and welfare of the public at large. State governments have broader powers than the federal government does, but when the federal government acts pursuant to one of its enumerated powers, the Constitution determines that federal law supersedes any conflicting state laws.

Because federal law supersedes conflicting state law, state laws regulating reproduction are constrained by federal protections for individual rights. But in the aftermath of the Supreme Court’s decision eliminating federal constitutional protection for the right to choose to terminate a pregnancy, there is considerable uncertainty about whether the Court will also overturn precedents protecting other forms of reproductive freedom, opening up even wider leeway for state restrictions and bans. The social conservativism of the current Congressional majority and the Trump administration raises the possibility of a new federal floor of restrictions on reproductive freedom. But for now, Congress and the Supreme Court have left the regulation of reproduction to the states. State legislatures and executive branch officials have adopted widely divergent approaches to regulating reproduction and federal priorities shift dramatically from election to election — setting up interstate and federal-state conflicts over reproductive justice.

## Interstate Conflicts Over Reproductive Justice

Some states have adopted restrictions on reproductive health care and surrogacy that align with religious directives about how children should be conceived, born, and raised. In other states, regulators defer to individual freedom and professional autonomy to make choices about family planning. In many states that defer to individual choice, legislators and executive branch officials have also taken action to protect individuals and institutions who provide reproductive health care from investigations, criminal prosecutions, and civil suits by out-of-state actors. Clashes between these states are intensifying.

Because choices about reproduction are made by multiple actors at multiple points in time and because these choices may be made in different geographic locations, state governments are limited in their legal authority and practical power to impose their will on the populace within their borders. A pregnant patient present in one state may be prescribed abortion medications by a clinician in another state, she may fill that prescription in a third location, ingest the medications in multiple locations over a period of several days, and pass the products of conception in yet another location. Similarly, an embryo may be created in one place using gametes collected in other locations, transferred to a patient’s uterus in one location, result in a live birth in another, and the resulting baby may be raised by non-birthing parents in yet another location pursuant to a legal agreement entered into by multiple parties who may not reside or execute the contract in the same jurisdiction. Interstate commerce and travel allow some degree of reproductive freedom for those who can afford it, even if they reside in restrictive states.^4^

Even as many states adopt harsh restrictions criminalizing health care that results in pregnancy termination, others are expanding state level protections for reproductive choice. In 2021, New York adopted legislation authorizing and regulating commercial surrogacy.^5^ In 2024, Michigan, which had previously been the only state with criminal prohibitions on surrogacy, adopted new legislation to decriminalize compensated surrogacy.^6^ As of late 2024, nearly half of states and the District of Columbia had adopted laws shielding individuals and organizations who provide protected forms of reproductive health care from civil and criminal liability and disciplinary action.^7^ State shield laws typically define protected reproductive health care broadly to include social and financial support, medications and health care services relating to contraception, assisted reproduction, pregnancy, termination of pregnancy, and miscarriage management.^8^

Law enforcement officials in states like Texas that restrict access to reproductive health care are on a collision course with officials in states like New York that not only permit pregnancy termination but shield those who provide it to patients residing in restrictive states.^9^ So far, conflicts have focused on pregnancy termination, along with parallel threats by restrictive state officials against out-of-state providers of gender-affirming care.^10^ But the mounting politicization of contraception and in vitro fertilization (IVF) raises the possibility that interstate disputes could expand to other reproductive realms in the future.

Restrictive states may be poised to create new hurdles for those seeking to build their families through assisted reproductive technologies and surrogacy, as signaled by the Alabama Supreme Court’s decision interpreting the state’s constitution to protect the rights of embryos as persons.^11^ The Alabama legislature responded by passing a measure to immunize assisted reproduction providers and patients from civil and criminal penalties for damaging or destroying embryos in the course of IVF treatment,^12^ but public advocacy was vital to this response and the outcome could be different in other states. If states do proceed with restricting IVF they may come into conflict with other states that protect it because those who can afford to do so will almost certainly travel across state lines to access care elsewhere.

## Federal-State Conflicts Over Reproductive Justice

Federal law fills gaps in state authority — over interstate commercial activities, for example — and takes precedence over conflicting state law in areas where federal and state powers overlap. State laws protecting access to reproductive health care services are vulnerable to federal override. Advocates for regulations that restrict reproductive choices are calling on the Trump Administration and Congress to use their power to impose federal restrictions that would supersede state protections for reproductive justice. Even if state laws protect reproductive rights, authorize pregnancy termination, or designate surrogacy contracts as enforceable in state courts, federal action to restrict reproductive services — especially when they cross state lines — would take precedence. If the federal administration revokes FDA approval for abortion pills or relies on the Comstock Act to prohibit interstate shipment of medications or devices used for reproductive health, that will take the issue away from the states. Similarly, if Congress chose to restrict assisted reproductive technologies or set parameters for surrogacy contracts based on their interstate effects, those laws would preempt contrary state laws.

## Does Science Matter When It Comes to Reproductive Justice?

In this rapidly changing legal and political environment, does research on the health and economic effects of access to contraception, abortion, assisted reproductive technology, or surrogacy still matter? When reproductive rights are constitutionally protected, scientific evidence can play an important role in legal disputes because judges typically apply a balancing test to determine whether a government intervention that burdens individual rights is justified by the government’s interest in mitigating harm. In an era when the courts have eliminated or eroded individual rights, leaving these matters to the democratic process, scientific evidence can have an impact if it sways the views of policymakers and their constituents. But in recent years, politicians and judges have repeatedly set aside carefully produced scientific evidence when it comes to matters of reproduction. Furthermore, scientific evidence plays a more limited role in disputes over which a government actor has power to regulate reproduction than it does in disputes over whether intrusions on reproductive freedom are constitutionally permissible.

On one hand, staunch opponents of reproductive choice are unlikely to be swayed by scientific evidence about health and economic effects. Although advocates for religious restrictions on reproductive freedom often point to negative health impacts as a justification for the restrictions they favor, it is widely understood that health impacts are not their real motivation. This phenomenon probably extends to other matters of reproduction as well. For example, concerns about economic exploitation of vulnerable surrogates may simply be a smokescreen for opposition to a mechanism that gay men rely on to become parents. If concerns about health and economic impacts are merely a smokescreen, research refuting those concerns may have little impact on those who most fervently oppose reproductive freedom.

As a legal matter, if concerns for negative health and economic effects associated with contraception, pregnancy termination, assisted reproductive technologies, or surrogacy were backed up by evidence, that could help religious advocates overcome legal protections for reproductive freedom, but advocates for reproductive restrictions based on religious beliefs will find it less and less necessary to rely on this kind of evidence as constitutional protections for reproductive autonomy are eroded and moral justifications are given more weight by the courts. In a pending case challenging the Trump administration’s order terminating transgender military servicemembers, the trial judge has indicated that animus against transgender people, by itself, appears to be the government’s sole justification for its harmful policy.^13^ The trial judge seems poised to enjoin the ban, but the Trump administration may fare better on appeal to the Supreme Court’s socially conservative majority. If the administration’s anti-trans interventions survive judicial scrutiny, opponents of LGBTQ rights could pursue restrictions on assisted reproduction or surrogacy based on similar animus, even in the absence of legitimate scientific evidence establishing harms.

On the other hand, the broader public and some judges may be persuadable. Animus and religious belief are politically unpopular as justifications for state restrictions on reproductive choices for individuals. In polls, white evangelical Protestants are the only religious group in which a majority support bans on abortion in all or most cases. In contrast, a majority of US residents who identify as Catholics support abortion being legal in all or most cases.^14^ Evidence countering anti-choice advocates’ claims of negative health and economic consequences could help lay bare the unscientific motivation behind restrictive regulations. Moreover, even if federal courts have retrenched constitutional protections for reproductive freedom, some state supreme courts are taking a different path and imposing state constitutional limits on state legislative and executive power to restrict reproductive choice. In addition, advocates for reproductive justice are crafting arguments based on equality and antidiscrimination to supplement or supplant arguments based exclusively on individual freedom. Evidence disputing harms supposedly caused by reproductive choices and technologies, evidence establishing harms caused by restrictions on reproductive choice, and evidence for the benefits of protections for reproductive choice still matters, even in the face of strong opposition from those who seek to stigmatize and restrict reproductive health care to further their religious directives, misogyny, and anti-LGBTQ animus.

## References

[1] Dobbs v. Jackson Women’s Health Org., 597 U.S. 215 (2022).

[2] D.S. Cohen, G. Donley, and R. Rebouché, “The New Abortion Battleground,” Columbia Law Review 123 no. 1 (2023): 1–100, at 2–3.

[3] L.F. Wiley and L.O. Gostin, Public Health Law & Ethics: Power, Duty, Restraint, 4th ed. (University of California Press 2025): 161.

[4] K. Drabiak, C. Wegner, V. Fredland, and P.R. Helft, “Ethics, Law, and Commercial Surrogacy: A Call for Uniformity,” Journal of Law, Medicine & Ethics 35 no. 2 (2007): 300–309 (arguing that federal regulation is needed because commercial surrogacy agencies to rely on the most supportive state laws while operating across state lines). 10.1111/j.1748-720X.2007.00139.x 17518856

[5] N.Y. Pub. Health L. § 2599-CC (2023); R. Klitzman, “Gestational Surrogacy, the Pope, and Needs for Regulations,” Journal of Law, Medicine and Ethics 53, no. 2: 221–226, 10.1017/jme.2025.66 (arguing that New York’s statute offers a robust model that additional states should adopt). 40432430

[6] Mich. Comp. L. § 722.1906 (2024).

[7] Center on Reproductive Health, Law, and Policy, “Shield Laws for Reproductive and Gender-Affirming Health Care: A State Law Guide,” UCLA Law, https://law.ucla.edu/academics/centers/center-reproductive-health-law-and-policy/shield-laws-reproductive-and-gender-affirming-health-care-state-law-guide (last updated September 2024) (describing shield law protections against civil liability adopted in seventeen states and the District of Columbia, all of which protect reproductive care and fifteen of which also apply to gender-affirming care).

[8] For example, see N.Y. Exec. Law § 837-x (2024) (defining “legally protected health activity” to include “supportive” services for “reproductive health,” including “all services, care and products relating to pregnancy, assisted reproduction, contraception, miscarriage management, or the termination of a pregnancy, and self-managed terminations”).

[9] M. Ziegler, “Texas Suit Against New York Doctor Ushers in New Era of Abortion Litigation,” *State Court Report*, January 14, 2025, https://statecourtreport.org/our-work/analysis-opinion/texas-suit-against-new-york-doctor-ushers-new-era-abortion-litigation.

[10] L.F. Wiley, “States as Shields,” Minnesota Law Review (forthcoming 2025).

[11] LePage v. Center for Reproductive Medicine, __ So.3d __, 2024 WL 656591 (Ala. 2024).

[12] Ala. Code § 6-5-810 (2024).

[13] “‘Unadulterated Animus’: Judge Exposes Inaccurate and Absurd Claims in Trump’s Executive Orders Targeting Transgender People,” GLAAD, February 19, 2025, https://glaad.org/judge-exposes-inaccurate-and-absurd-claims-in-trumps-executive-orders-targeting-transgender-people/.

[14] PRRI Staff, “Public Opinion on Abortion” Pew Research Center, May 13, 2024, https://www.pewresearch.org/religion/fact-sheet/public-opinion-on-abortion/; “Views on LGBTQ Rights in All 50 States: Findings from PRRI’s 2023 American Values Atlas,” PRRI, March 12, 2024, https://www.prri.org/research/views-on-lgbtq-rights-in-all-50-states/.

